# Eyelid Infestation: A Case Report of Atypical Phthiriasis Palpebrarum

**DOI:** 10.7759/cureus.25383

**Published:** 2022-05-27

**Authors:** Renato Correia Barbosa, Rita Basto, Ana Rita Viana, Alexandre Reis Silva, Ricardo Bastos

**Affiliations:** 1 Ophthalmology Department, Hospital Pedro Hispano - Unidade Local de Saúde de Matosinhos, Matosinhos, PRT

**Keywords:** parasitosis, blepharitis, eyelash, eyelid, phthirus pubis

## Abstract

Phthiriasis palpebrarum is a rare parasitosis of the eyelashes caused by *Phthirus* *pubis*. This report describes an atypical case of this disease.

A 72-year-old female patient suffered prolonged symptoms of severe left eye pruritus for 18 months, refractory to conventional eyelid hygienic measures, and anti-histaminic and corticosteroid medications. Slit-lamp examination showed multiple translucent oval structures adherent to the upper eyelashes, and 18 crab-like lice, which were mechanically removed and characterized as *Phthirus pubis*. Treatment was started with corticosteroid and antibiotic ointment, vaseline, and Blephademodex® wipes (Laboratoires Théa, Auvergne-Rhone-Alpes, France). After 2 weeks, all symptoms had subsided completely.

Although rare, phthiriasis palpebrarum may be easily confused with frequent palpebral pathologies like blepharitis. A careful slit-lamp examination is central for proper evaluation and diagnosis. Mechanical removal of the lice is the most effective treatment but should be complemented by topical and/or systemic treatment. This report presented an atypical case of this disease.

## Introduction

Phthiriasis palpebrarum or phthiriasis ciliaris is a rare parasitosis of the eyelashes caused by *Phthirus pubis*, also known as crab louse or pubic louse, an arthropod that is an obligate ectoparasite of humans [1]. It is an insect that belongs to the *Phthiridae* family, 2 mm long, with a crab-like round body, thick sets of legs, and large claws, which allow it to adhere to human hair. A female louse lays an average of three nits daily, which hatch 7 to 10 days later [2-5]. Its primary habitat is pubic hair, and the most typical route of transmission is through sexual intercourse. Other means of transmission, such as indirect transmission through clothes, towels, or contaminated objects are uncommon, and their plausibility is still unclear. *Phthiriasis pubis *infestation is commonly associated with poor hygiene and overcrowding, and is most prevalent in sexually active patients, generally between 15 and 45 years old [6].

The purpose of this work is to report an atypical case of this disease, diagnosed in a 72-year-old woman. Data were collected from the clinical records of the patient.

## Case presentation

A 72-year-old female patient presented in a first appointment, referred from primary care, with prolonged symptoms of severe left eye pruritus, for at least 18 months, refractory to conventional eyelid hygienic measures, topical and oral anti-histaminic medication, and topical corticosteroid drops or ointments.

The patient was well-kept, reported adequate hygiene care, and lived in an environment with guaranteed sanitary conditions. She reported no current or recent sexual activity, and symptoms were unilateral for the whole duration. She did not have a history of red eye, eye pain, visual acuity loss, or other ophthalmological complaints. During the ophthalmologic examination, she had a best-corrected visual acuity of 20/20 in both eyes, and intraocular pressure of 16/17 in her right and left eye, respectively. Unaided examination revealed a quiet eye without apparent signs of inflammation.

**Figure 1:**
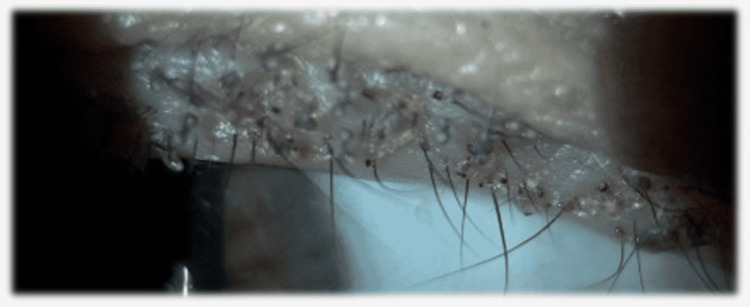
Slit-lamp photography of the eyelid margin before lice and nit removal.

**Figure 2:**
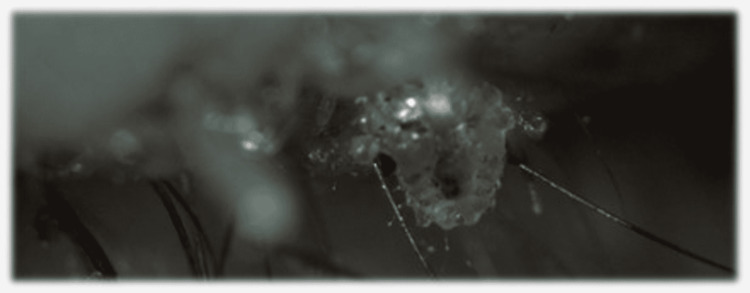
Slit-lamp photography of lice adherent to the palpebral margin.

**Figure 3:**
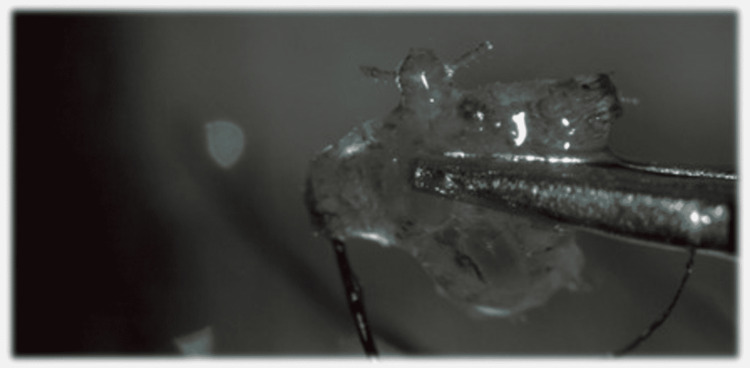
Slit-lamp close-up photography of lice after mechanical removal.

Slit-lamp examination showed multiple translucent oval structures were found adherent to the eyelashes of the upper left eyelid, almost entirely covering them. She presented excoriation of the outer margin of the eyelid and brownish scales. Additionally, 18 crab-like lice were found, mechanically removed, and inspected using the biomicroscope (Figures 1-3).

They were characterized as *Phthirus pubis*, which confirmed the diagnosis of phthiriasis palpebrarum. Following the removal of all lice, treatment was commenced with corticosteroid and antibiotic ointment, vaseline, and Blephademodex® eyelid wipes (Laboratoires Théa, Auvergne-Rhone-Alpes, France). She was also referred to the dermatology department, where the involvement of the remaining body surface was excluded, as well as other dermatological conditions. After 2 weeks, in a follow-up consultation, all her symptoms had subsided completely.

## Discussion

Although rare, phthiriasis palpebrarum may be easily confused with more frequent palpebral pathology, as it mimics symptoms described in common palpebral diseases such as blepharitis, chalazion, or hordeolum [6-8]. Pruritus of the eyelids is the main symptom and is due to a cutaneous hypersensitivity reaction towards the louse saliva.

Like most other ophthalmic conditions, a careful slit-lamp examination is required for proper evaluation and diagnosis. The small size and translucent characteristics of the parasite and its nits make them barely visible to the naked eye. Mechanical removal of the lice is the most effective treatment option, but should always be complemented by topical and/or systemic treatment. Several modalities of treatment are described. Parasympathomimetic agents, such as physostigmine or pilocarpine, fluoresceine, and liquid petrolatum ointment (Vaseline) have all been reported to be efficient. Topical antiparasitic agents such as pyrethrins, pyrethroids, or lindane can also be prescribed. Oral ivermectin can be used as a single dose treatment, but a second dose may be necessary after 10 days to control the newly hatched nits. However, oral ivermectin is contraindicated in children younger than five and pregnant or lactating women [9]. In case of infestation of other parts of the body, treatment is mandatory. All sexual contacts and family members should be evaluated for the presence of palpebral or pubic infestation. Given the frequency of sexual transmission, patients should be screened for other sexually transmitted infections. A diagnosis in a child should lead the physician to rule out the possibility of sexual abuse [10].

Two features that make this case atypical were the patient's age and the prolonged unilateral involvement. Contrary to this report, this disease typically affects children, adolescents, and young adults. A description of seven cases of the disease by Anane et al. showed a mean age of 22 years, with the oldest patient being 50 years old [5]. Since in elderly patients there are much more prevalent pathologies that may present similar symptoms, such as blepharitis, a low degree of suspicion is usually attributed, which may delay the diagnosis, or make it more difficult. Another atypical characteristic of our case was the fact that the patient maintained unilateral symptoms for as long as 18 months. In the majority of the reports, this disease has bilateral involvement. When unilateral cases are found, they are usually associated with a shorter symptomatic course, as an initially unilateral involvement tends to become bilateral, due to the extension of the risk factors that led to the infection of the first eyelid [1, 2, 3, 6, 8].

## Conclusions

This report presented an atypical case of unilateral phthiriasis palpebrarum which is otherwise known as a bilateral disease. Unilateral cases are uncommon, especially in a prolonged setting, with active symptoms for 18 months.

Careful slit-lamp examination is paramount to avoid misdiagnosis, especially in chronically symptomatic cases, which do not resolve after conventional blepharitis treatment. A high level of suspicion is recommended, even in the absence of typical risk factors involving inadequate hygiene care or sexual risk factors.
